# Constructing Quasi‐Localized High‐Concentration Solvation Structures to Stabilize Battery Interfaces in Nonflammable Phosphate‐Based Electrolyte

**DOI:** 10.1002/advs.202411826

**Published:** 2024-12-16

**Authors:** Chenyang Shi, Mengran Wang, Zari Tehrani, Bo Hong, Mengnan Wang, Rui Tan, Serena Margadonna, Yanqing Lai, Maria Magdalena Titirici

**Affiliations:** ^1^ School of Metallurgy and Environment Central South University Changsha Hunan 410083 China; ^2^ Engineering Research Centre of Advanced Battery Materials The Ministry of Education Changsha Hunan 410083 China; ^3^ National Energy Metal Resources and New Materials Key Laboratory Changsha Hunan 410083 China; ^4^ National Engineering Research Center of Advanced Energy Storage Materials Changsha Hunan 410083 China; ^5^ Department of Chemical Engineering Swansea University Swansea SA1 8EN UK; ^6^ Department of Chemical Engineering Imperial College London London SW7 2AZ UK; ^7^ Hunan Province Key Laboratory of Nonferrous Value‐Added Metallurgy Central South University Changsha Hunan 410083 China

**Keywords:** battery safety, flame‐retardant electrolytes, fluorinated phosphates, molecular design, solvation shell tuning

## Abstract

Flame‐retardant phosphate‐based electrolytes effectively enhance lithium‐ion battery safety but suffer from poor compatibility with graphite anodes and high‐voltage cathodes, hindering scalability. Fluorinated phosphates, though widely used, increase interfacial resistance at the anode, degrading performance. In this work, carbonate solvents with strong polarity are introduced to prevent tris(2,2,2‐trifluoroethyl) phosphate (TFEP) from participating in the solvation structure of lithium ions. This strategy forms a quasi‐localized high‐concentration solvation structure, thereby restricting the reduction of TFEP and its impact on the graphite anode. The LiNi_0.8_Mn_0.1_Co_0.1_O_2_ (NCM811) | Graphite (Gr) pouch cell with optimized electrolyte exhibits a capacity retention rate of 80.1% after 370 cycles at 0.5C, which is much more stable than the electrolyte with TFEP‐involved solvation structure (capacity retention rate: 47.1% after 300 cycles). The corresponding pouch cell with cut‐off voltage to 4.5 V exhibits a capacity retention rate of 82.8% after 125 cycles, significantly outperforming cells employing commercial carbonate electrolytes (capacity retention rate: 56.9% after 125 cycles). Thus, the developed quasi‐localized high‐concentration solvation structure can effectively stabilize the electrode interface, greatly enhancing the cycling performance of phosphate‐based flame‐retardant electrolytes.

## Graphical abstract

By introducing strongly polar carbonates to construct a localized high‐concentration solvation structure with tris(2,2,2‐trifluoroethyl) phosphate (TFEP) as a diluent, compatibility between nonflammable phosphate‐based electrolyte and the electrodes is achieved resulting in excellent electrochemical performance while ensuring safety.

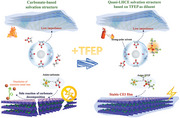

## 1 Introduction

Currently, lithium‐ion batteries play an indispensable role in 3C digital devices, electric vehicles, energy storage, and other fields.^[^ ^1^, ^2^, ^3^ ^]^ However, safety incidents involving lithium‐ion batteries utilizing carbonate electrolytes frequently occur, posing significant challenges for their further deployment.^[^ ^4^, ^5^ ^]^ Enhancing the safety of electrolytes has emerged as the focal point of research.^[^ ^6^, ^7^, ^8^ ^]^ Adding flame‐retardant solvents into carbonate electrolyte can improve battery safety while maintaining the cost‐effectiveness.^[^ ^9^ ^]^ Among various solvents, phosphate solvents have emerged as the preferred choice due to their high flame‐retardant efficiency, low cost and strong ability for dissolving lithium salts.^[^ ^10^ ^]^ However, the high molecular polarity of phosphate could strongly interact with lithium ions,^[^ ^11^ ^]^ leading to co‐intercalation with lithium ions into graphite anode, which deteriorates the electrochemical performance of batteries.^[^ ^12^, ^13^ ^]^ Moreover, the low oxidation potential of phosphates makes it difficult to match with high‐voltage cathode. Currently, fluorination has been widely reported to effectively increase the oxidation potential of phosphates and reduce the binding energy between phosphates and lithium ions.^[^ ^14^, ^15^, ^16^ ^]^ Nevertheless, fluorophosphate‐based electrolytes cannot form a stable solid electrolyte interface (SEI) film. Fluorophosphate‐moieties involved in SEI can decompose on the anode surface, increasing interfacial resistance, and undesirably diminishing electrochemical performance.^[^ ^17^, ^18^ ^]^ Given these challenges, it is crucial to minimize the impact of fluorophosphate on the anode interface to enhance electrochemical performance and ensure safety.

To stabilize the anodic interface of fluorophosphate, various strategies are being explored. The current strategy is to introduce components that are preferentially reduced over tris(2,2,2‐trifluoroethyl) phosphate (TFEP) at the anode to form the SEI film, such as introducing film‐forming additives and using a high‐concentration lithium salts electrolyte.^[^ ^19^, ^20^, ^21^, ^22^ ^]^ However, the issue of poor stability in TFEP reduction has not been resolved.^[^ ^23^, ^24^ ^]^ With ongoing cycling, it undergoes reduction and decomposition at the anode, leading to increased resistance in the SEI film. As reported, organic molecules involved in the solvation structure exhibit a preferential reduction potential and tend to form SEI films.^[^ ^25^, ^26^ ^]^ Thus, by preventing fluorophosphate from integrating into the lithium‐ion solvation structure, its reduction at the anode could be effectively inhibited, thus promoting the formation of an SEI film originated from ethylene carbonate (EC) and achieving both electrolyte safety and electrochemical cycling stability.

In this work, a novel quasi‐localized high‐concentration solvation structure, is proposed to mitigate the impact of fluorophosphate on electrochemical performance. When TFEP is introduced into the EC, DMC (dimethyl carbonate)‐based carbonate electrolyte whose polarity is higher than TFEP, TFEP will remain freely in the outer layer of the lithium ion solvation structure like a “diluent” effectively enhancing the reduction stability of TFEP in the electrolyte. The NCM811|Gr pouch cell with strong polarity carbonate solvents has a capacity of 161 mAh after 370 cycles at 0.5C, and its capacity retention rate reaches 80.2%. Upon increasing the charging cut‐off voltage to 4.5 V, the pouch cell exhibits a capacity retention rate of 82.8% after 125 cycles, significantly outperforming cells employing carbonate electrolytes (capacity retention rate: 56.9% after 125 cycles). Furthermore, this design concept effectively promotes the application of phosphate‐based flame‐retardant electrolytes.

## 2 Results and Discussion

**Figure 1:**
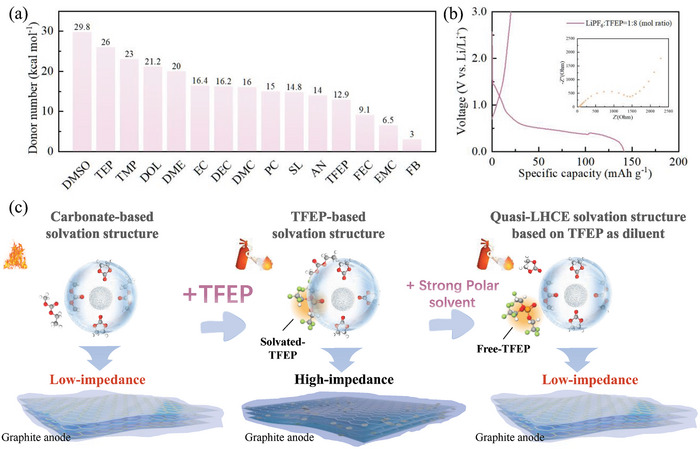
a) Donor number of different solvents. b) Initial charge and discharge curves of Li|graphite half batteries in LiPF_6_‐TFEP electrolytes with Li^+^‐TFEP molar ratio at 1:8. c) Schematic diagram of the influence of different electrolytes on the interface.

Current commercial electrolytes typically utilize carbonate solvents with varying polarities to maintain the electrochemical properties of batteries, such as EC/DMC/EMC (Ethyl Methyl Carbonate) blends. Despite the significantly lower polarity of fluorophosphate, such as TFEP, compared to triethyl phosphate (TEP) (the donor number (DN) value reduced from 26 to 12.9), it remains higher than certain carbonate solvents, as depicted in **Figures** **1**a and S1, Supporting Information.^[^ ^27^, ^28^, ^29^ ^]^ This leads to fluorophosphate replacing a portion of the weakly polar carbonate solvent to engage in the solvation structure of lithium ions, and due to its significantly reduced reduction stability, leading to the decomposition of TFEP on the anode surface (Figure S2, Supporting Information). TFEP decomposes at the graphite anode (Figure 1b), and its decomposition products result in the battery impedance to increase significantly to 1500 Ω. Building upon this, it is proposed that replacing a portion of the weakly polar carbonate solvent with a carbonate possessing a polarity stronger than TFEP may effectively prevent TFEP from engaging in the solvation structure of lithium ions. This will result in the formation of a solvation structure resembling that of a localized high‐concentration electrolyte: polar carbonate molecules will appear in the inner layer of the lithium ion solvation structure, while TFEP, limited by its weaker polarity, will reside in the outer layer of the solvation structure, akin to a diluent molecule in the localized high‐concentration electrolyte.^[^ ^30^ ^]^ This quasi‐localized high‐concentration solvation structure prevents the decomposition of fluorophosphate molecules at the graphite anode, ensuring that the electrochemical performance of the battery remains stable as shown in Figure 1c. Therefore, in conjunction with DN values and the binding energy (Figure S3, Supporting Information) between solvents and lithium ions, DMC (DN: 16), EMC (DN: 6.5), and methyl 2,2,2‐trifluoroethyl carbonate (FEMC) whose binding energy (−0.02632 eV) is significantly lower than other solvents, have been chosen as the research subjects to investigate the influence of solvents with varying polarities on the solvation structure.^[^ ^31^ ^]^

**Figure 2:**
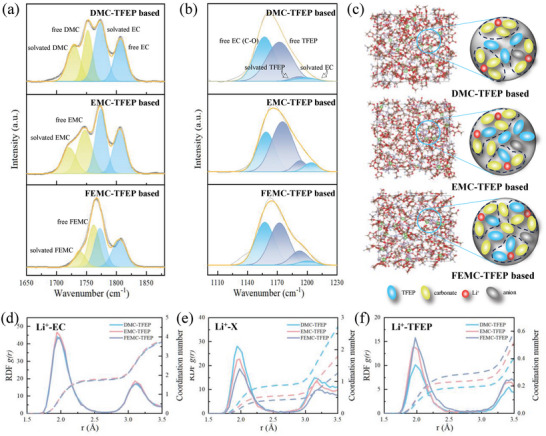
FTIR spectra of a,b) DMC‐TFEP (1.5 M LiPF_6_ EC/DMC/TFEP = 3/3/4 vol ratio), EMC‐TFEP (1.5 M LiPF_6_ EC/EMC/TFEP = 3/3/4 vol ratio) and FEMC‐TFEP (1.5 M LiPF_6_ EC/FEMC/TFEP = 3/3/4 vol ratio)‐based electrolytes in different regions. c) Schematic diagram of solvation structures of different electrolytes, Radial distribution functions (RDF) and coordination number of d) Li‐O_EC_, e) Li‐O_DMC,EMC.FEMC_, and f) Li‐O_TFEP_ pairs.

To validate that strong polar solvents inhibit TFEP from entering solvation structures, FTIR tests were conducted on various electrolytes. As illustrated in **Figure** **2**a, the peak at 1806 cm^−1^ represents free EC molecules, while the characteristic peak of coordinated‐EC molecules is observed at 1772 cm^−1^.^[^ ^32^ ^]^ Upon comparison of various electrolytes, it can be observed that EC molecules participate in the solvation structure under similar conditions. Conversely, the characteristic peaks of DMC, EMC, and FEMC in the 1730–1769 cm^−1^ range suggest unequal involvement of these solvents in solvation structures. Analysis of the peak area ratio reveals that DMC exhibits the highest proportion of participation in the solvation structure among different electrolyte formulations (the peak at 1749 cm^−1^ corresponds to free DMC molecules, while the peak at 1721 cm^−1^ represents DMC molecules involved in the solvation structure).^[^ ^33^ ^]^ Conversely, FEMC, with a weaker binding ability to lithium ions, predominantly exists in the electrolyte in a free state.^[^ ^34^ ^]^ Moreover, the FTIR results illustrate the status of TFEP (Figure 2b). The peak at 1172 cm^−1^ represents P = O of the free TFEP, while the peak at 1192 cm^−1^ corresponds to coordinated‐TFEP. Comparative analysis of TFEP across different electrolytes reveals that TFEP in DMC‐TFEP electrolyte primarily exists in free form, with minimal participation in the solvation structure of lithium ions. Conversely, the amount of TFEP involved in solvation structure in the EMC‐TFEP electrolyte is notably higher than that in the DMC‐TFEP electrolyte. Furthermore, the quantity of coordinated‐TFEP in the FEMC‐TFEP electrolyte is further increased. These results are generally consistent with previous assumptions indicating that the presence of a highly polar carbonate leads to its participation in the lithium ion solvation structure, occupying space and reducing the possibility of TFEP participating in the solvation structure. Furthermore, molecular dynamics simulations have been utilized to further investigate the distribution of solvent molecules in different electrolytes. In Figure 2d, the RDF and corresponding coordination number of EC molecules around lithium ions in different electrolytes are presented. The participation of EC in the solvation structure of lithium ions across different electrolytes remains consistent, with nearly identical RDF and coordination numbers. As depicted in Figure 2e, the coordination number of carbonate molecules in the lithium‐ion solvation structure gradually decreases with decreasing molecular polarity, and DMC exhibits a significantly higher coordination number than EMC and FEMC. Consequently, the content of TFEP in the solvation structure exhibits an opposite trend, with the coordination number of TFEP participating in the solvation structure in the FEMC‐TFEP electrolyte being about twice that in the DMC‐TFEP electrolyte (Figure 2f). Additionally, the number of anions involved in the solvation structure is relatively small. Furthermore, across different electrolyte systems, there is no significant change in the number of anions participating in the solvation structure (Figure S4, Supporting Information). As shown in the Figure S5, Supporting Information, in the DMC‐TFEP, EMC‐TFEP, and FEMC‐TFEP electrolyte systems, the ^19^F signal gradually shifts to lower fields. This also suggests a reduction in the electron cloud density around the fluorine nuclei, indicating an increased involvement of TFEP in the solvation structure of lithium ions. As shown in Figure 2c, DMC with stronger polarity exhibits a stronger tendency to bind with lithium ions compared to EMC and FEMC with weaker polarity than TFEP, thereby preventing TFEP from participating in the solvation structure of lithium ions and forming a quasi‐localized high concentration electrolyte solvation structure with TFEP as the “diluent.”

**Figure 3:**
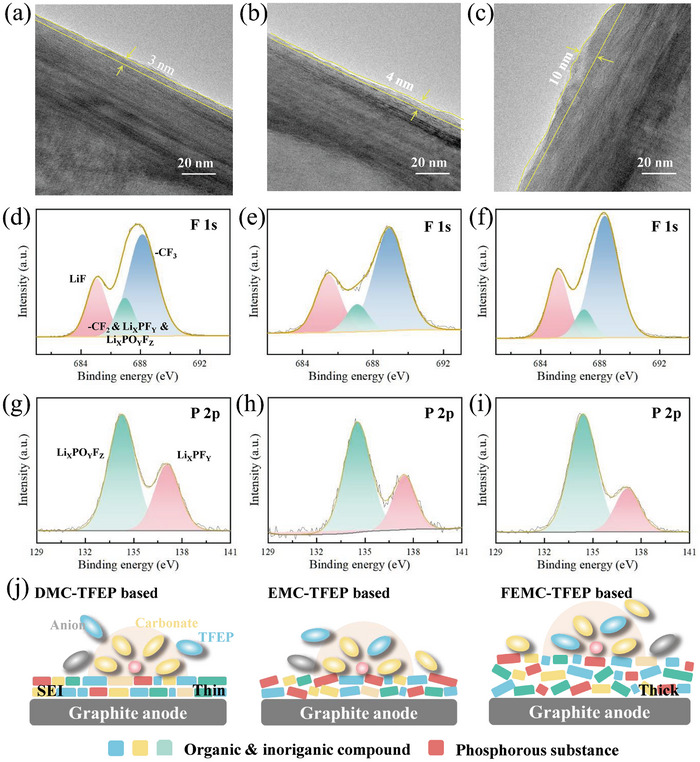
Transmission Electron Microscopy (TEM) images of graphite electrodes after 3 cycles with a) DMC‐TFEP, b) EMC‐TFEP and c) FEMC‐TFEP electrolytes. Surface passivation chemistry on graphite anodes after 3 cycles at 0.1C given by X‐ray Photoelectron Spectroscopy (XPS) results of F 1s and P 2p in d,g) DMC‐TFEP, e,h) EMC‐TFEP and f,i) FEMC‐TFEP electrolytes. j) Schematic diagram of SEI films with different electrolytes.

To investigate the impact of various electrolytes on the graphite anode, the Gr|Li half‐cell was assembled and the graphite anode after 3 cycles was tested. The Scanning Electron Microscopy (SEM) results (Figure S6, Supporting Information) indicate that different electrolytes do not damage the structure of the graphite anode. Nevertheless, the SEI film thickness of the graphite anode with DMC‐TFEP electrolyte is ≈2–3 nm, with a relatively uniform distribution as depicted in **Figure** **3**a. Conversely, with EMC‐TFEP electrolyte, the SEI film thickness is about 4 nm (Figure 3b), while with FEMC‐TFEP electrolyte, it exceeds 10 nm with uneven thickness distribution (Figure 3c). In instances where TFEP does not participate in the solvation structure of lithium ions, the SEI film appears thin and uniform. However, with a higher proportion of TFEP in the solvated structure, the SEI film becomes thicker and exhibits uneven distribution. This increase in SEI film thickness significantly raises battery resistance, consequently deteriorating electrochemical performance. In addition, XPS tests were performed on the activated graphite anode. As shown in Figure 3d, the primary decomposition products on the graphite anode with DMC‐TFEP electrolyte include LiF (685.6 eV)^[^ ^35^ ^]^ produced by lithium salt decomposition, and ‐CF_3_ (689.2 eV) produced by TFEP decomposition.^[^ ^24^ ^]^ The presence of ‐CF_3_ on the anode surface significantly increases with EMC‐TFEP electrolyte (Figure 3e), suggesting a more severe TFEP decomposition compared to the DMC‐TFEP electrolyte. Furthermore, as depicted in Figure 3f, the ‐CF_3_ content on the surface of the graphite anode with FEMC‐TFEP electrolyte exhibits further increases. Regarding the P 2p spectra (Figure 3g–i), LiP_x_F_y_ and Li_x_PO_y_F_z_ are presumed to be the decomposition products of salt and carbonate,^[^ ^36^ ^]^ with the primary decomposition product of TFEP being Li_x_PO_y_F_z_.^[^ ^37^ ^]^ With the diminishing inhibitory effect of DMC, EMC, and FEMC on preventing TFEP from participating in the lithium‐ion solvation structure, the content of Li_x_PO_y_F_z_ gradually increases, suggesting a corresponding escalation in TFEP decomposition on the graphite anode. Figure 3j demonstrates the influence of various electrolytes on the SEI film. TFEP decomposition on the anode with DMC‐TFEP electrolyte is constrained, resulting in a thin and compact SEI film. However, employing FEMC as a co‐solvent with weaker polarity enhances TFEP decomposition, resulting in the formation of a thick and uneven SEI film.

**Figure 4:**
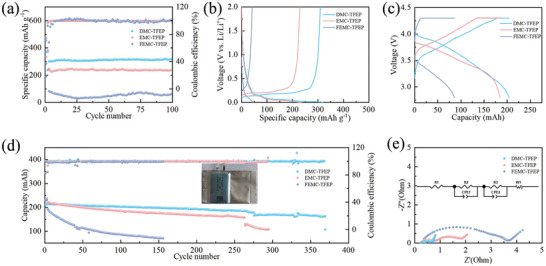
Electrochemical performance of Gr|Li at 0.5C with a) different electrolytes and the b) corresponding 50^th^ charging discharging curves. The performance of NCM811|Gr pouch cells at 0.5 C with d) different electrolytes and the c) corresponding 100^th^ charging discharging curves. e) The EIS of pouch cells with different electrolytes after cycles.

In order to verify the impact of various electrolytes on electrochemical performance, the Gr|Li battery was tested. As shown in **Figure** **4**a, the battery with DMC‐TFEP electrolyte exhibits an initial specific capacity of 306.5 mAh g^−1^, and no significant decrease in capacity is observed after 100 cycles. In contrast, batteries with EMC‐TFEP electrolyte exhibit an initial discharge specific capacity of only 246.5 mAh g^−1^, indicating a significant decrease compared to batteries with DMC‐TFEP electrolyte. Moreover, the capacity of the battery with FEMC‐TFEP electrolyte decreased even more significantly. This could be attributed to an excessive decomposition of TFEP on the graphite anode. Furthermore, the CV curve in Figure S7, Supporting Information illustrates that the FEMC‐TFEP electrolyte induces a notable decrease in the capacity of the graphite anode during the initial cycle. Figure 4b depicts the charge‐discharge curve of the 50th cycle of the half cell. In comparison to the charge‐discharge curve of the first cycle (Figure S8, Supporting Information), the battery with EMC‐TFEP or FEMC‐TFEP electrolyte shows a substantial increase in polarization voltage after 50 cycles. In addition, 200 mAh pouch cells were assembled with different electrolytes to further verify the effect on the graphite anode. As shown in Figure 4d, the pouch cell with DMC‐TFEP electrolyte has a capacity of 161 mAh after 370 cycles at 0.5C, and its capacity retention rate reaches 80.1%. In contrast, the capacity degradation of the cell with EMC‐TFEP electrolyte is more rapid. After 300 cycles, the cell shows a significant capacity degradation, and the capacity retention rate was 47.1%. The capacity of the pouch cell with FEMC‐TFEP electrolyte decays more rapidly, with a capacity retention of 30.1% after 150 cycles, which may be due to the continuous decomposition of TFEP causing the rapid increase in impedance, leading to rapid capacity degradation. Moreover, the charge‐discharge curve of the 100th cycle indicates that the polarization of the cell with DMC‐TFEP electrolyte is lower than that of cells with EMC‐TFEP or FEMC‐TFEP electrolyte (Figure 4c). Furthermore, the charging capacity of the pouch cell with DMC‐TFEP electrolyte is primarily facilitated by the rate charging process. These findings suggest that the polarization of cells utilizing DMC‐TFEP electrolyte is markedly lower than that of cells with EMC‐TFEP or FEMC‐TFEP electrolyte (Figure S9, Supporting Information). At the same time, compared to other electrolyte formulations based on TFEP or phosphate solvents, batteries using DMC‐TFEP electrolyte also demonstrate superior electrochemical performance as shown in Table S1, Supporting Information. Last, electrochemical impedance tests have been performed on pouch cells with different electrolytes after cycles as shown in Figure 4e and Table S2, Supporting Information. The impedance of cells containing EMC (R_SEI_ = 0.176 Ω) is nearly three times higher than that of cells containing DMC (R_SEI_ = 0.564 Ω), while the impedance of cells containing FEMC (R_SEI_ = 1.416 Ω) is nearly eight times higher than that of DMC‐containing cells. Additionally, NCM811|Gr coin full cells (NCM811: 8.3 mg cm⁻^2^, Gr: 5.8 mg cm⁻^2^) were assembled to evaluate their electrochemical performance at varying temperatures and cutoff voltages. The results show that, under different conditions, the cells using the DMC‐TFEP electrolyte exhibited the best electrochemical performance (Figure S10, Supporting Information). These results suggest that an excess of TFEP participates in the solvation structure of lithium ions, leading to its continued decomposition in the graphite anode, which consequently results in increased impedance and significant degradation in electrochemical performance. This observation indicates that TFEP continues to decompose on the graphite anode as the cycle progresses, thereby causing an increase in impedance and contributing to a notable deterioration in battery performance.

**Figure 5:**
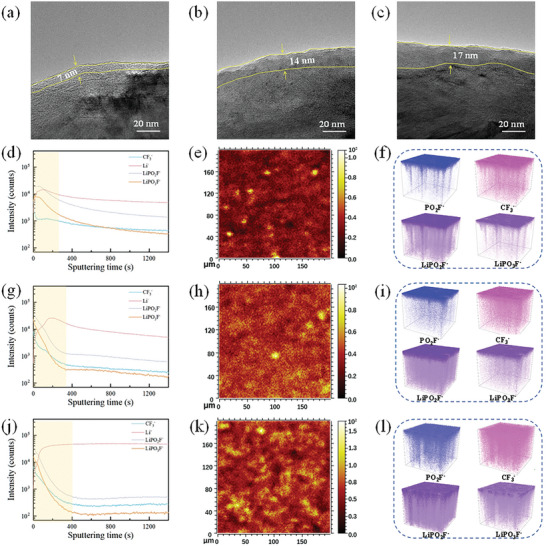
TEM of graphite electrodes after cycles with a) DMC‐TFEP, b) EMC‐TFEP, and c) FEMC‐TFEP electrolytes. Normalized ToF‐SIMS intensity depth profiles of surface and bulk fragments composing the anode‐electrolyte interphase with the d) DMC‐TFEP, g) EMC‐TFEP and j) FEMC‐TFEP electrolytes. Overlay 2D images constructed by summation over all sputter times showing LiPO_3_F^−^ secondary ions on the graphite electrode surface with the e) DMC‐TFEP, h) EMC‐TFEP and k) FEMC‐TFEP electrolytes. 3D visualization of selected various secondary‐ion fragments on the graphite anodes when being cycled with the f) DMC‐TFEP, i) EMC‐TFEP and l) FEMC‐TFEP electrolytes are given by the TOF‐SIMS characterization.

To further investigate the impact of various electrolytes on SEI films, the anode was characterized after cycling. As illustrated in **Figure** **5**a, the SEI film thickness of the graphite anode after cycling with DMC‐TFEP electrolyte measures ≈7 nm, exhibiting a relatively uniform distribution. Conversely, the SEI film thickness with EMC‐TFEP electrolyte measures ≈14 nm, while with FEMC‐TFEP electrolyte, it reaches 17 nm, significantly thicker than that observed with DMC‐TFEP electrolyte (Figure 5b). And as observed from the SEM results of the anode surface, the surface coverage with EMC‐TFEP and FEMC‐TFEP electrolytes appears to be more substantial compared to that with DMC‐TFEP electrolyte (Figure S11, Supporting Information). These results indicate that the increased participation of TFEP in the solvation structure leads to more severe side reactions. To investigate the distribution of different components, the graphite anode after cycling was analyzed using TOF‐SIMS, as shown in Figure 5d–l. The decomposition products of TFEP mainly manifest as PO_2_F^−^, CF_3_^−^, LiPO_2_F^−^, and LiPO_3_F^−^. From the depth profile plot of TOF‐SIMS (Figure 5d,g,j), it can be observed that the composition of the anode surface with DMC‐TFEP electrolyte does not change after ≈250 s of etching, which can be considered as the thickness of the outer layer SEI film. In comparison, electrodes using EMC‐TFEP and FEMC‐TFEP electrolytes stabilize their composition only after ≈350 and 400 s of etching, respectively, indicating a thicker SEI film on their electrode surfaces. Additionally, the content of Li^−^ (considered to be dead lithium and lithium‐containing compounds) secondary ions on the anode surface gradually increases in DMC‐TFEP, EMC‐TFEP, and FEMC‐TFEP electrolytes, indicating a gradual exacerbation of side reactions between the electrolyte and the anode. Furthermore, by comparing the concentration of LiPO_3_F^−^ on the SEI film, considered to be the main product of TFEP decomposition, it further elucidates the decomposition of TFEP on the anode. The total LiPO_3_F^−^ fragments on the surface were found to be 5.11 × 10^5^ total counts in DMC‐TFEP electrolyte (Figure 5e) while the value were 7.03 × 10^5^ and 1.06 × 10^6^ total counts in EMC‐TFEP and FEMC‐TFEP electrolyte, respectively (Figure 5h,k). This shows that the content of TFEP decomposition products on the surface of SEI film gradually increases. The 3D visualization of selected various secondary‐ion fragments displays a detailed composition of the SEI film. As shown in Figure 5f, it can be observed that the decomposition products of TFEP are primarily distributed on the outer layer of the SEI film with the DMC‐TFEP electrolyte, and as the etching depth increases, the decomposition products of TFEP gradually decrease. This indicates that in the early stages of cycling, the SEI film is formed by the decomposition of EC. As cycling progresses, a small amount of TFEP decomposes and accumulates on the surface of the SEI film. In contrast, the decomposition products of TFEP with the EMC‐TFEP electrolyte penetrate deeper compared to those with the DMC‐TFEP electrolyte, indicating the occurrence of TFEP decomposition in the initial stages of cycling (Figure 5i). However, the decomposition products of TFEP are distributed almost throughout the structure of the SEI film with the FEMC‐TFEP electrolyte (Figure 5l). This indicates a more severe decomposition of TFEP, which is also the fundamental reason for battery failure. This observation further suggests that as TFEP participates in the lithium‐ion solvation structure, its decomposition on the anode surface persists throughout the cycling process, leading to a continuous rise in battery impedance and a rapid deterioration in its electrochemical performance. Additionally, the cathode materials after cycling were characterized to investigate the impact of different electrolytes on the stability of the cathode structure. As shown in the Figure S12a–c, Supporting Information, the CEI film on the cathode materials using EMC‐TFEP and FEMC‐TFEP electrolytes is noticeably thicker compared to those using the DMC‐TFEP electrolyte. At the same time, cross‐sectional SEM images show that the cathode material with the DMC‐TFEP electrolyte retains its structural integrity after cycling (Figure S12d, Supporting Information), while the surface of the cathode with EMC‐TFEP and FEMC‐TFEP electrolytes exhibits partial damage Figure S12e,f, Supporting Information. This suggests that the DMC‐TFEP‐based cells maintain superior cathode structure after extended cycling, likely due to the excessive consumption of active lithium at the anode, which leads to the collapse of the cathode structure in the comparison groups. The electrolyte after cycling was analyzed using NMR, and it was found that no significant changes occurred, except for a slight shift in the peak positions, which may be due to alterations in the solvation structure during cycling. This suggests that TFEP remains stable throughout the entire cycling process (Figure S13, Supporting Information).

**Figure 6:**
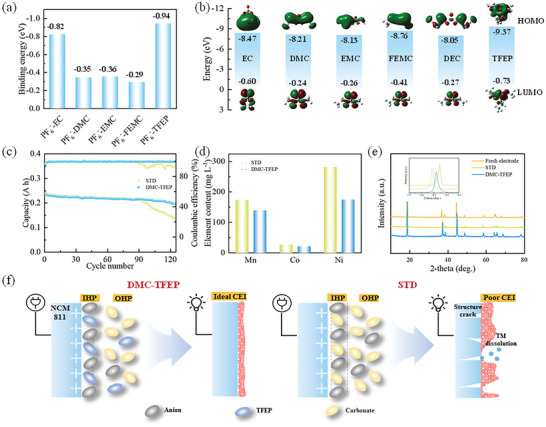
a) The interaction between PF_6_^−^ and different solvents. b) Frontier molecular orbital energy levels (HOMO and LUMO) of various solvents. c) The performance of NCM811|Gr pouch cells at 0.5 C with different electrolytes at a 4.5 V charging cut‐off voltage. d) The dissolved TMs‐ion concentration in the cycled STD (1 M LiPF_6_ EC/DEC/EMC = 1:1:1 vol ratio) and DMC‐TFEP electrolyte. e) X‐ray diffraction (XRD) patterns of the NCM811 before and after cycles in STD and DMC‐TFEP electrolyte. The enlarged XRD patterns show the shift of the (003) peak. f) The influence of TFEP and carbonate with different anion‐solvent interaction on the cathode's IHP structure and the formation of cathode electrolyte interphase (CEI).

In addition, the high‐voltage performance of DMC‐TFEP electrolyte has been also studied. The stability of the CEI film is typically dictated by the anions and solvent molecules that bind to them. Solvents possessing a strong positive electrostatic potential tend to preferentially associate with anions, accumulating on the cathode surface due to the influence of electric field forces, and subsequently decomposing to generate the CEI film. Therefore, the strength of the interaction between different solvent molecules and anions in the electrolyte has been theoretically calculated first. As shown in **Figures** **6**a and S14, Supporting Information, it is evident that EC has a significantly stronger binding ability with PF_6_^−^ compared to other carbonate solvents. Consequently, EC accumulates alongside anions on the cathode surface, subsequently undergoing continuous decomposition in the standard electrolyte (STD) electrolyte. This constitutes the fundamental reason why the present carbonate electrolyte fails to sustain a stable cycle at high voltage. In contrast, the binding energy of TFEP and PF_6_^−^ is −0.94 eV, which is higher than the binding energy of EC and PF_6_^−^ (−0.82 eV). This shows that TFEP is preferentially combined with PF_6_^−^ and accumulates on the cathode surface under the action of electric field force and its high oxidation stability effectively slows down the decomposition of solvents on the cathode surface (Figure 6b). In addition, the linear sweep voltage results also show that the oxidation potential of DMC‐TFEP electrolyte reaches 5.05 V, which is much higher than the 4.65 V of STD electrolyte (Figure S15, Supporting Information). Pouch cells are assembled separately with different electrolytes and the charging cut‐off voltage rises to 4.5 V. As shown in Figure 6c, the capacity retention rate of the pouch cell with DMC‐TFEP electrolyte after 125 cycles is 82.8%. Conversely, the capacity of the pouch cell with STD electrolyte rapidly decreases after 90 cycles, and the Coulombic efficiency also shows a significant decrease. The capacity retention rate of the pouch cell is 56.9% after 125 cycles, indicating that the side reactions of electrolyte oxidation are more severe. In addition, the capacity of pouch cells with EMC‐TFEP and FEMC‐TFEP electrolytes shows rapid degradation at initial cycles, due to the decomposition of TFEP on the anode (Figure S16, Supporting Information). As shown in Figure S17a, Supporting Information, the charging and discharging curves of the pouch cell with the STD electrolyte exhibit significant decay after 120 cycles. Meanwhile, cells with EMC‐TFEP and FEMC‐TFEP electrolytes show rapid decay during the initial cycles (Figure S17c,d, Supporting Information). In contrast, the cell with the DMC‐TFEP electrolyte shows no significant degradation (Figure S17b, Supporting Information). Additionally, the charge‐discharge curves reveal that the polarization of cells with EMC‐TFEP and FEMC‐TFEP electrolytes is more pronounced compared to the DMC‐TFEP cell. Subsequently, the pouch cells after cycles have been disassembled and the electrodes have been characterized subsequently. The ICP test of the graphite anode after cycling shows that the content of transition metal (Ni/Co/Mn) in the anode with STD electrolyte is significantly higher than that with DMC‐TFEP electrolyte (Figure 6d). This indicates that under a cut‐off voltage of 4.5 V, the cathode undergoes structural collapse, leading to the dissolution of transition metals. Additionally, continual surface reactions during the cycle induce the H2‐H3 phase transition and (003) peak shifts as shown in Figure 6e, which suppress the intercalation/deintercalation of lithium ions.^[^ ^38^ ^]^ Additionally, SEM results reveal that NCM particles with STD electrolyte exhibit significant cracking compared to those with DMC‐TFEP electrolyte (Figure S18, Supporting Information). TEM results show that the CEI film with STD electrolyte is thicker and more uneven compared to that with DMC‐TFEP electrolyte, which is believed to result from the continuous decomposition of carbonate on the cathode surface (Figure S19, Supporting Information). The XPS results also indicate that the cathode surface with STD electrolyte is mainly composed of ROCO_2_Li and Li_X_PF_Y_ decomposed from carbonate and lithium salts, while the cathode with DMC‐TFEP electrolyte is mainly composed of LiF (Figure S20, Supporting Information). These results indicate that free TFEP molecules have the potential to replace carbonate molecules in binding with PF₆⁻ and accumulate on the cathode surface, effectively improving the oxidation stability of the electrolyte and enhancing the stability of the cathode material under high voltage. Additionally, the adsorption of TFEP‐PF₆⁻ on the NCM811 surface probably alter spin polarization, suppress malignant phase transitions, and improve CEI film properties (Figure 6f).

**Figure 7:**
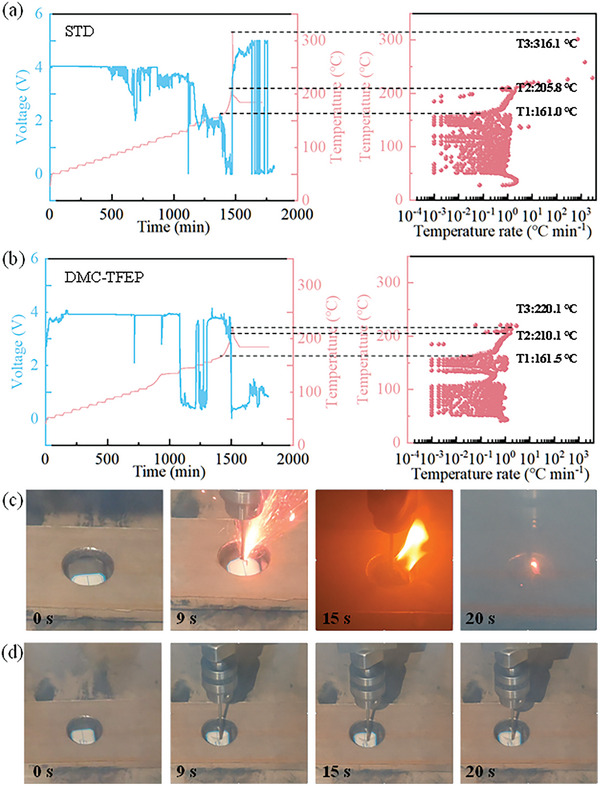
Safety features of 600 mAh NCM811|Gr pouch cells. a) Cells with the STD electrolyte. b) Cells with the DMC‐TFEP electrolyte. Optical photos of pouch cells in c) STD and d) DMC‐TFEP electrolyte in the pre‐mid‐late stages of needling.

The accelerating rate calorimetry (ARC) was used to investigate the safety characteristics of 600 mAh NCM811|Gr practical cells with various electrolytes. As depicted in **Figure** **7**a,b, both cells demonstrate comparable self‐heating temperatures (T1). With increasing temperature, the cell employing STD electrolyte reached thermal runaway at 205.8 °C (T2), whereas the DMC‐TFEP electrolyte cell reached thermal runaway ≈210.1 °C. Subsequently, the temperature rise rate of the cell with STD electrolyte sharply rose to 2963.5 °C min⁻¹, reaching a maximum temperature of 316.1 °C (T3). In contrast, the cell with DMC‐TFEP electrolyte exhibited a temperature rise rate of only 219.5 °C min^−1^ after thermal runaway, reaching a maximum temperature of 220.1 °C. This indicates that the incorporation of TFEP substantially reduced heat release after thermal runaway in the batteries. Furthermore, the nail penetration test is conducted using 600 mAh NCM811|Gr pouch cells with STD and DMC‐TFEP electrolyte to further prove the safety performance. In the initial stage, batteries using STD electrolyte exhibit noticeable volume expansion, possibly due to the generation of a large amount of gas. Subsequently, the battery ruptures, releasing a significant amount of smoke and ejecting flames. As the combustion progresses, the battery gradually turns into charcoal as shown in Figure 7c. In stark contrast, batteries using DMC‐TFEP electrolyte show no structural changes throughout the puncture process and no combustion phenomenon, indicating superior safety performance of this electrolyte (Figure 7d).

## 3 Conclusion

In this work, a quasi‐localized high‐concentration solvation structure was designed by tuning the solvent polarity to reduce the adverse impact of fluorinated phosphate on the graphite anode, addressing its poor reduction stability, resulting in enhanced battery cycling stability and high‐voltage performance while ensuring safety. The NCM811|Gr pouch cell demonstrates a capacity retention rate exceeding 80% after 370 cycles. Upon increasing the charging cut‐off voltage to 4.5 V, the pouch cell exhibits a capacity retention rate of 82.8% after 125 cycles, significantly outperforming cells employing carbonate electrolytes. Furthermore, this design concept offers a novel approach for utilizing solvents with moderate polarity, yet limited compatibility with the graphite anode, in electrolytes. In the future, employing techniques such as Electrochemical Quartz Crystal Microbalance (EQCM)^[^ ^39^ ^]^ to monitor changes in the SEI film could provide a more precise evaluation of the compatibility between the solvation structure and the electrode.

## Conflict of Interest

The authors declare no conflict of interest.

## Supporting information



Supporting Information

## Data Availability

The data that support the findings of this study are available in the supplementary material of this article.
