# Supplementary material for: Constructing Quasi‐Localized High‐Concentration Solvation Structures to Stabilize Battery Interfaces in Nonflammable Phosphate‐Based Electrolyte
Source: Adv Sci (Weinh). 2024 Dec 16;12(6):2411826. doi: 10.1002/advs.202411826 (PMC11809392; doi:10.1002/advs.202411826)
Supplement: Supplementary file 1 — Supporting Information [file ADVS-12-2411826-s001.docx]

**Supporting information**

Constructing quasi-localized high-concentration solvation structures to stabilize battery interfaces in nonflammable phosphate-based electrolyte

Chenyang Shi ^a^, Mengran Wang ^a,b,d,e^*, Zari Tehrani ^f^, Bo Hong ^a,b,d,e^*, Mengnan Wang ^g^, Rui Tan ^f^*, Serena Margadonna ^f^, Yanqing Lai^a,c,d,e^, Maria Magdalena Titirici ^g^

^a^ School of Metallurgy and Environment, Central South University, Changsha 410083, Hunan, China

^b^ Engineering Research Centre of Advanced Battery Materials, The Ministry of Education, Changsha 410083, Hunan, China

^c^ Hunan Province Key Laboratory of Nonferrous Value-Added Metallurgy, Central South University, Changsha 410083, Hunan, China

^d^ National Energy Metal Resources and New Materials Key Laboratory, Changsha, 410083, Hunan, China

^e^ National Engineering Research Center of Advanced Energy Storage Materials, Changsha, 410083, Hunan, China

^f^ Department of Chemical Engineering, Swansea University, SA1 8EN, Swansea, UK.

^g^ Department of Chemical Engineering, Imperial College London, SW7 2AZ, London, UK

***Correspondence.**

E-mail address: [mengranwang93@163.com](mailto:mengranwang93@163.com); bop_hong@csu.edu.cn; rui.tan@swansea.ac.uk

**Experiment**

Electrolyte preparation: Battery-grade ethylene carbonate (EC), dimethyl carbonate (DMC), ethyl methyl carbonate (EMC), methyl trifluoroethyl carbonate (FEMC), tris(2,2,2-trifluoroethyl) phosphate (TFEP),and lithium hexafluorophosphate (LiPF_6_) were provided by DoDoChem, China. The STD electrolyte was 1 M lithium hexafluorophosphate (LiPF_6_) in ethylene carbonate (EC)/ diethyl carbonate (DEC)/ ethyl methyl carbonate (EMC) (v/v/v = 1:1:1). Electrolytes with different carbonate (DMC, EMC and FEMC) were prepared by adding 0.228g LiPF_6_ into the EC/carbonate/TFEP (v/v/v=3/3/4) solution. All electrolytes were prepared in the glovebox where the H_2_O and O_2_ is strictly controlled below 0.1 ppm.

Electrode preparation and cell assembly: Artificial graphite (Dongguan Kaijin New Energy Technology Co., Ltd., China), carbon black (AB), and polyvinylidene difluoride (PVDF, Ofluorine Chemical Tech Co., Ltd., China) were dried at 60 ºC under vacuum condition before use. The graphite electrode was prepared by mixing artificial graphite, carbon black, and PVDF in a weight ratio of 80%: 10%: 10%, and then coating onto the Cu foil. The obtained electrode was dried at 80 ºC for 1 h, and then dried at 120 ºC under vacuum condition. The diameter of electrode is 12 mm and the mass loading of active material is about 1~2 mg/cm^2^ for the anode. Celgard PE separators were used in Li/Gr half-cells with STD, DMC-TFEP, EMC-TFEP, and FEMC-TFEP electrolytes. By adding 1.5g of electrolyte to the 200 mAh pouch cell provided by Lifang new power company, its electrochemical performance in the pouch cell was tested.

Electrochemical measurements: Linear sweep voltammetry was performed on a CHI600C Electrochemical Analytical Instrument (Chenhua) in selected electrolytes to measure the electrochemical stability windows by using a microelectrode with a stainless steel sheet as working electrode and a Li foil as both reference and counter electrodes. The scanning rate is 1 mV/s and the voltage ranges from OCV to 6 V. The cyclic voltammetry experiments of the battery were performed at a scan rate of 0.1 mV s^−1^ between 0 V to 2 V. All charge/discharge tests were conducted on LAND system at room temperature. Li/graphite cells with STD, DMC-TFEP, EMC-TFEP and FEMC-TFEP electrolytes were discharged and charged at 0.5 C (1 C = 372 mAh) in the potential range of 0.005-3 V. Graphite/NCM811 pouch cells with different electrolytes were charged/discharged at 0.1 C for 3 cycles and at 0.5 C for subsequent cycles between 2.5 and 4.3 V.

Electrode characterization: The morphological images of the graphite electrodes before and after cycling in different electrolytes were obtained using scanning electron microscopy (FESEM, ZEISS Merlin Compact VP) and Transmission Electron Microscope (Talos F200X S/TEM). The cycled batteries were disassembled in a glove box under argon atmosphere. The cycled graphite electrodes were harvested and rinsed with anhydrous dimethyl carbonate to remove residual solvents, followed by vacuum drying at room temperature (25 °C) for SEM and TEM characterization. Xray photoelectron spectroscopy (XPS, ESCALAB250, USA.) was used to analyze the composition of the electrode surface. X-ray diffraction (XRD, Bruker D8 ADVANCE, Germany) was conducted to determine the structural change of active materials after cycling and Rietveld refinement was performed using Topas software. A TOF-SIMS 5 instrument (ION-TOF, Münster, Germany) was used for TOF-SIMS depth profiling. The sputtering beam was Cs+ working at 1 keV.

Computational details: First-principles calculations based on density functional theory were performed in Gaussian (G16) suite of program with Becke's three parameter hybrid method using the Lee–Yang–Parr correlation functional (B3LYP). The geometrical structures and the vibrational modes were calculated at 6-31+G(d) level. The binding energy (E_b_) between two components was defined as following:

𝐸_b_=𝐸_total_-𝐸_A_ -𝐸_B_

where E_total_, E_A_, and E_B_ are the total energy of the A–B complexes, A component, and B component, respectively. A and B can be Li ion, anions, and solvents.

Theoretical IR vibrations were obtained using Gaussian 16 at the B3LYP/6–31++G(d) theoretical level. The calculated IR data were then accumulated to obtain the total spectrum for each electrolyte. The adiabatic reduction potentials for the representative solvation structures were calculated using the following function.

E_adiabatic_=-(G _reduced_ -G _initial_+ΔG_solv_^0^ (reduced)- ΔG_solv_^0^ (initial))/ F− 1.4V

where G_reduced_ and G_inital_ are the free energies of the reduced and initial complexes at 298.15 K in gas-phase at 298.15 K, respectively; ΔG_solvo_ are the corresponding free energies of solvation, and F is the Faraday constant. The zero-point energy (ZPE) corrections were considered in the calculation while the basis set superposition error (BSSE) energy was neglected. The solvent effects in the free energy calculation of each complex were considered using an implicit polarizable continuum model (PCM). The spin density calculation of the reduced state structures was conducted using natural bond orbital (NBO) theory. Ulteriorly, DFT-D3 dispersion correction is adopted to make the results more accurate.

MD Simulation: MD simulations were performed in LAMMPS and the all-atom optimized potentials for liquid simulations (OPLS-AA) force field was used to describe molecules/ions interactions between molecules/ions in this work. The initial structures were built with the salt and solvent molecules distributed in the simulation boxes using Moltemplate. Besides, the particle-mesh Ewald method was used to evaluate the long-range electrostatic interactions with a grid spacing of 1.2 Å. Firstly, the electrolyte systems were equilibrated in the constant temperature (300 K) and constant pressure (NPT ensemble) for 3 ns before finally being subjected to 5 ns of constant volume and constant temperature dynamics (NVT ensemble). Then the Radial distribution functions were obtained by the pymatgen code.

Safety tests: Flame tests of different electrolytes were performed by directly igniting the electrolytes. 0.2 g of the electrolyte was dropped into a circular stainless-steel shell with 20 mm in diameter. Then, the electrolyte was ignited with N-butane blowtorch at an ignition temperature of approximately 800 °C. The intensity of the electrolyte combustion was recorded by photography and video. The Accelerating Rate Calorimeter (ARC) tests were conducted with an EV+ ARC system purchased from Thermal Hazard Technology. For each ARC test, two fully charged 600mAh batteries were combined with a thermocouple in between. The starting temperature was 40℃ with a heating step of 5℃. The detected self-heating rate was 0.02℃/min and the waiting time was 40 min. The nail penetration test of pouch cells is evaluated using Battery Nail Penetration Tester (BE‒8110) with the nail size of ɸ5*100 mm and the speed of 25 mm/s. Before testing, the cells are full charged.


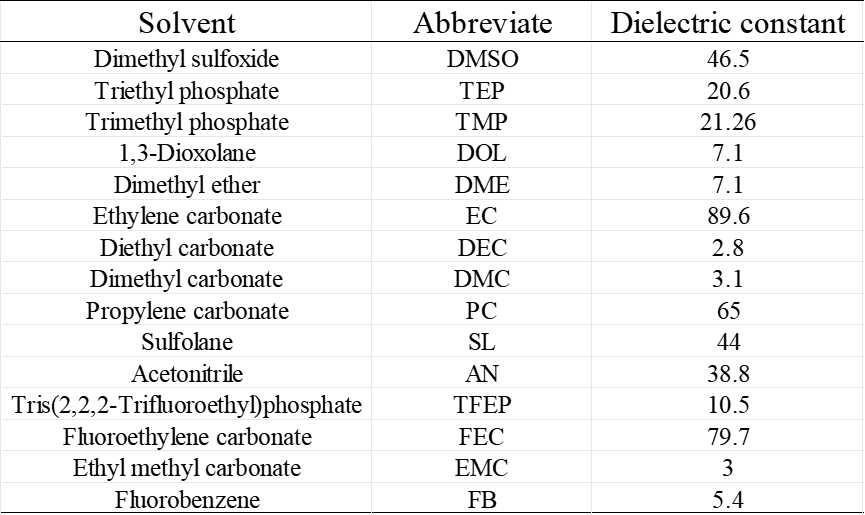


Fig S1. Different solvents and their corresponding abbreviations.

Fig S2. Frontier molecular orbital energy levels (HOMO and LUMO) of various solvents.

Fig S3. (a) the interaction between Li^+^ and different solvents and the corresponding structures (b).


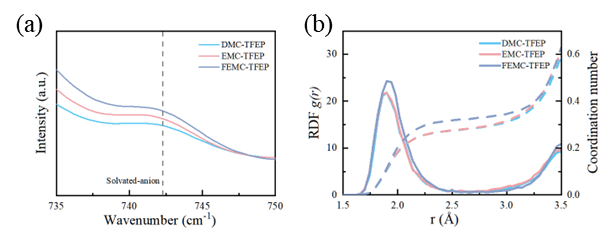


Fig S4. (a) FTIR spectras of different electrolytes between 735-750 cm^-1^. (b) Radial distribution functions and coordination number of Li^+^-PPF_6_^-^ pairs.


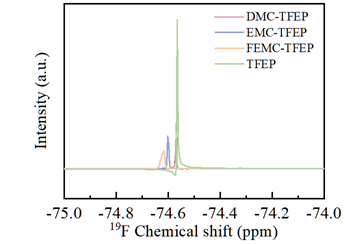


Fig S5. ^19^F NMR spectroscopy of different electrolyte.

Fig S6. SEM images of graphite electrodes after 3 cycles with DMC-TFEP (a), EMC-TFEP (b) and FEMC-TFEP (c) electrolytes.

Fig S7. Initial CV curves of graphite electrodes with different electrolytes.

Fig S8. The initial charging discharging curves of Gr|Li batteries with different electrolytes


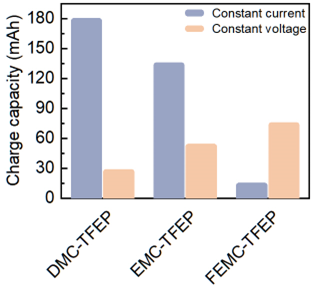


Fig S9. Capacity of constant current charging and constant voltage charging.


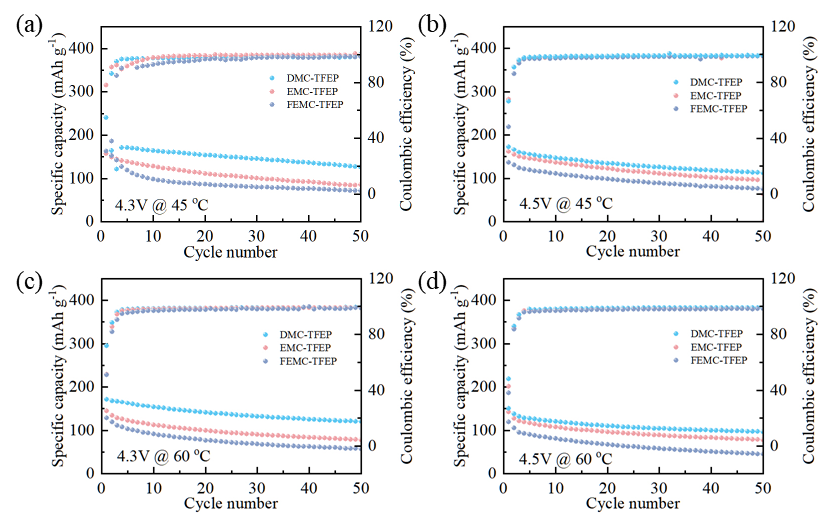


Fig S10. The performance of NCM811|Gr coin cells at 0.5 C with different electrolytes at 45 and 60 oC with (a, c) 4.3 V and (b, d) 4.5V charging cut-off voltage.

Fig S11. SEM images of graphite electrodes after cycles with DMC-TFEP (a), EMC-TFEP (b) and FEMC-TFEP (c) electrolytes.


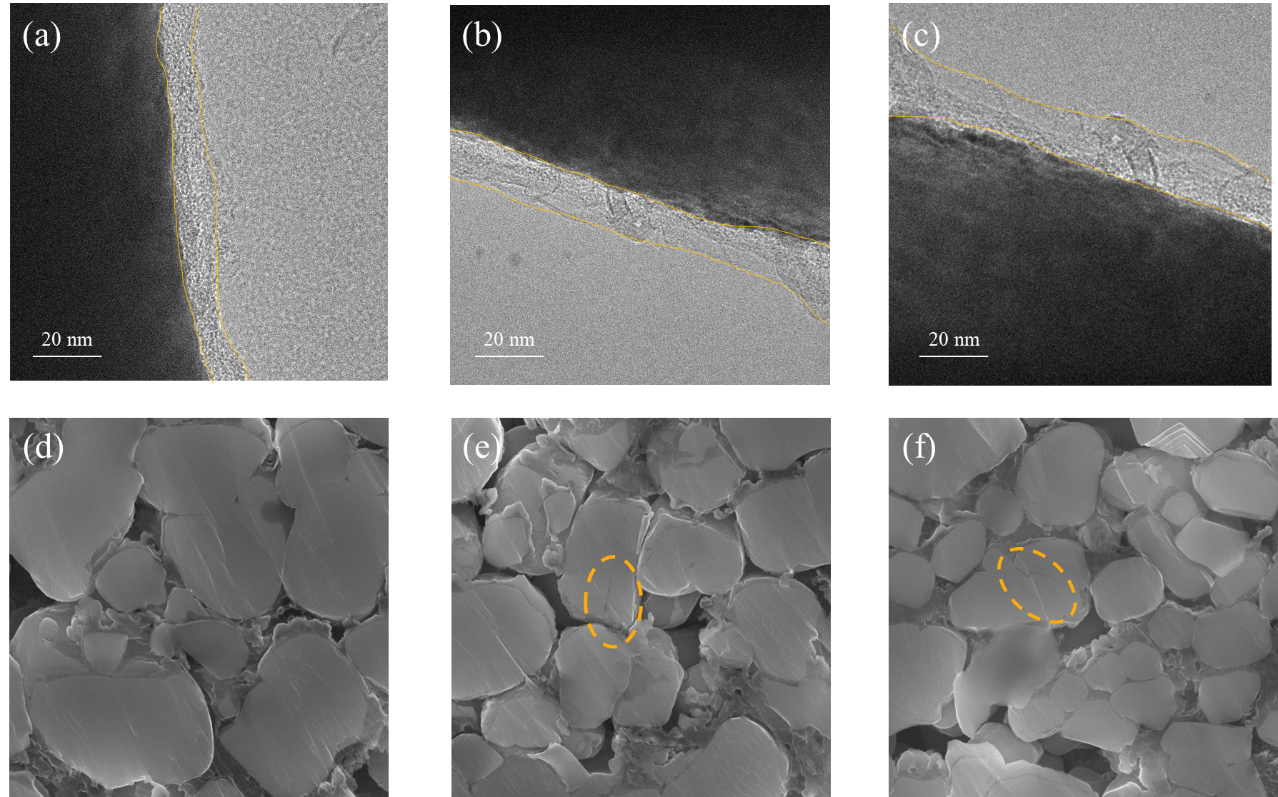


Fig S12. TEM images of the NCM811 cathode electrodes after cycling with DMC-TFEP (a), EMC-TFEP (b), and FEMC-TFEP (c) electrolytes. Cross-sectional SEM images of the NCM811 cathode electrodes after cycling with DMC-TFEP (d), EMC-TFEP (e), and FEMC-TFEP (f) electrolytes.


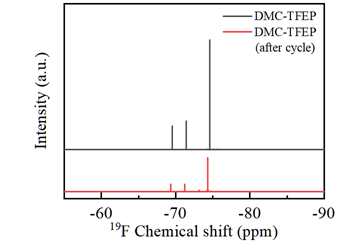


Fig S13. ^19^F NMR spectroscopy of DMC-TFEP electrolyte before and after cycles.

Fig S14. The structures combing PF_6_^-^ and different solvents.

Fig S15. Linear sweep voltammetry of the STD and DMC-TFEP electrolyte at a sweep rate of 1 mV.


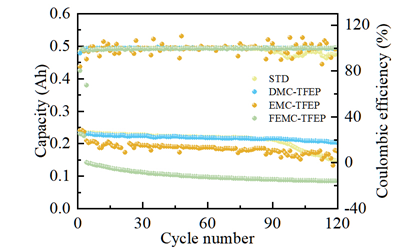


Fig S16. The performance of NCM811|Gr pouch cells at 0.5 C with different electrolytes at a 4.5 V charging cut-off voltage.


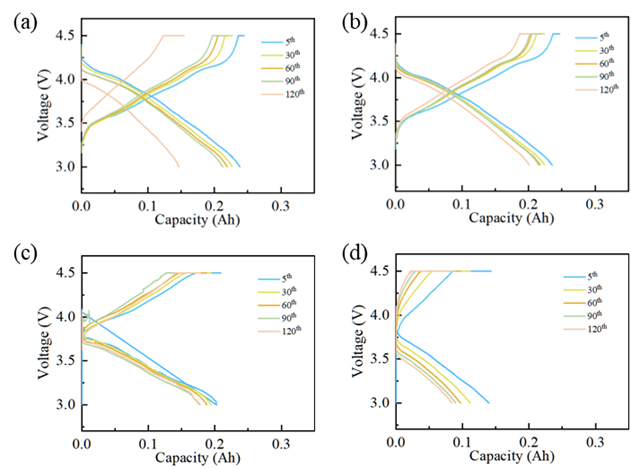


Fig S17. The charging discharging curves of NCM811|Gr pouch cells at 0.5 C with STD (a); DMC-TFEP (b); EMC-TFEP (c) and FEMC-TFEP (d) electrolytes.


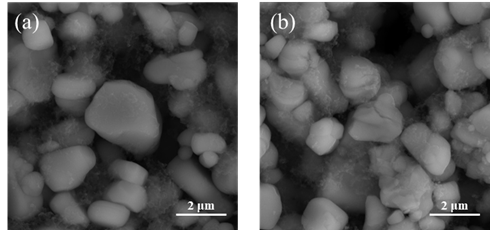


Fig S18. SEM images of NCM811 after cycles with DMC-TFEP (a) and STD (b) electrolytes.

Fig S19. TEM images of NCM811 after cycles with DMC-TFEP (a) and STD (b) electrolytes.

Fig S20. Surface passivation chemistry on NCM811 after cycles at a 4.5V cutoff voltage given by XPS results of F 1s and Li 1s in STD (a, c) and DMC-TFEP (b, d) electrolytes.

**Table S1.** Properties of representative nonflammable LEs and their applications in LIBs.

| Electrolytes | Flammability limits | Battery type | Cycles (capacity retation) | Ref |
| --- | --- | --- | --- | --- |
| 1M LiPF_6_ EC/DEC/TFEP 3.5/3.5/3 (vol ratio) | No ignition | NCM622\|Gr | 500 (41.2%) | ^1^ |
| 1M LiPF_6_ GBL/TFEP 7/3 (vol ratio) +3% LiODFB | No ignition | NCM622\|Gr | 500 (13.3%) | ^1^ |
| 1M LiPF_6_ FEMC/TFEP 4/6 (vol ratio) + 5 vol% FEC + 0.1 M LiODFB | No ignition | NCM811\|Gr | 150 (80%) | ^2^ |
| 1M LiPF_6_ EC/DEC/TFEP 3.5/3.5/3 (wt ratio) | No ignition | NCM111\|Gr | 50 (81.5%) | ^3^ |
| 3.3 M LiFSA TFEP | No ignition | LiNi_0.8_Co_0.15_Al_0.05_O_2_\|Gr | 10 (85%) | ^4^ |
| LiFSI-TEP (1:2 mol ratio) + 5 vol% FEC + 0.05 M LiBOB | No ignition | LiCoO_2_\|Gr | 50 (90%) | ^5^ |
| LiPF_6_/TEP/EC/EMC (1:2:3:4 mol ratio) | No ignition | LiNi_0.65_Co_0.15_Mn_0.2_O_2_\|Gr | 120 (67.7%) | ^6^ |
| LiFSI:TMP:TTFEP (1:1.4:1 mol ratio) | No ignition | LFP\|Gr | 300 (71.5%) | ^7^ |
| LiFSI:TMP:EC:TTFEP  (1:1.2:0.2:1 mol ratio) | No ignition | LFP\|Gr | 300 (79.9%) | ^7^ |
| 1.5M LiPF_6_ EC/DMC/TFEP 3/3/4 (vol ratio) | No ignition | NCM811\|Gr | 370 (80.7%) | This paper |

TEP: Triethyl phosphate; TMP: trimethyl phosphate; TTFEP: tris(2,2,2-trifluoroethyl) phosphite

Table S2. Model´s parameters for fitted spectra.

| Electrolyte | R_SEI_ | R_ct_ |
| --- | --- | --- |
| DMC-TFEP | 0.176 | 0.228 |
| EMC-TFEP | 0.564 | 1.131 |
| FEMC-TFEP | 1.416 | 1.757 |

References

1. Gebert, F.; Longhini, M.; Conti, F.; Naylor, A. J., An electrochemical evaluation of state-of-the-art non-flammable liquid electrolytes for high-voltage lithium-ion batteries. *J. Power Sources* **2023,** *556*.

2. Chen, L.; Shen, X.; Chen, H.; Wen, T.; Rao, R.; Zhang, C.; Meng, Q.; Zhang, J.; Ding, Y.; Ai, X.; Cao, Y.; Chen, Z., High-stable nonflammable electrolyte regulated by coordination-number rule for all-climate and safer lithium-ion batteries. *Energy Storage Materials* **2023,** *55*, 836-846.

3. Murmann, P.; Mönnighoff, X.; von Aspern, N.; Janssen, P.; Kalinovich, N.; Shevchuk, M.; Kazakova, O.; Röschenthaler, G.-V.; Cekic-Laskovic, I.; Winter, M., Influence of the Fluorination Degree of Organophosphates on Flammability and Electrochemical Performance in Lithium Ion Batteries: Studies on Fluorinated Compounds Deriving from Triethyl Phosphate. *J. Electrochem. Soc.* **2016,** *163* (5), A751-A757.

4. Shiga, T.; Kato, Y.; Kondo, H.; Okuda, C.-a., Self-extinguishing electrolytes using fluorinated alkyl phosphates for lithium batteries. *Journal of Materials Chemistry A* **2017,** *5* (10), 5156-5162.

5. Zeng, Z.; Murugesan, V.; Han, K. S.; Jiang, X.; Cao, Y.; Xiao, L.; Ai, X.; Yang, H.; Zhang, J.-G.; Sushko, M. L.; Liu, J., Non-flammable electrolytes with high salt-to-solvent ratios for Li-ion and Li-metal batteries. *Nature Energy* **2018,** *3* (8), 674-681.

6. Liu, M.; Zeng, Z.; Gu, C.; Ma, F.; Wu, Y.; Wu, Q.; Yang, X.; Chen, X.; Cheng, S.; Xie, J., Ethylene Carbonate Regulated Solvation of Triethyl Phosphate to Enable High-Conductivity, Nonflammable, and Graphite Compatible Electrolyte. *ACS Energy Letters* **2023,** *9* (1), 136-144.

7. Jia, H.; Yang, Z.; Xu, Y.; Gao, P.; Zhong, L.; Kautz, D. J.; Wu, D.; Fliegler, B.; Engelhard, M. H.; Matthews, B. E.; Broekhuis, B.; Cao, X.; Fan, J.; Wang, C.; Lin, F.; Xu, W., Is Nonflammability of Electrolyte Overrated in the Overall Safety Performance of Lithium Ion Batteries? A Sobering Revelation from a Completely Nonflammable Electrolyte. *Advanced Energy Materials* **2022,** *13* (4).
